# Evidence-Based Interventions for Functional Restoration and Pain Management following Traumatic Brain Injury

**Published:** 2026-04-06

**Authors:** Sugeeth Kandikattu, Manas Aavula, David Parvizi, Devendra K Agrawal

**Affiliations:** Department of Translational Research, College of Osteopathic Medicine of the Pacific, Western University of Health Sciences, Pomona, California 91766 USA

**Keywords:** Chronic pain, Cognitive rehabilitation, Concussion, Functional impairment, Neurorehabilitation, Pain management, Physical therapy, Rehabilitation, Traumatic brain injury

## Abstract

Traumatic brain injury is a major cause of death and long-term disability worldwide and can lead to persistent functional impairment and chronic pain that significantly impact quality of life. Patients with traumatic brain injury commonly experience motor deficits, impaired balance and coordination, cognitive dysfunction, and behavioral changes. Chronic pain, particularly post-traumatic headaches and musculoskeletal pain, is also frequently reported and can limit participation in rehabilitation and daily activities. This narrative literature review summarizes current evidence on the epidemiology, pathophysiology, and clinical course of traumatic brain injury, with a focus on functional restoration and pain management. Existing research supports the use of multidisciplinary rehabilitation approaches that incorporate physical therapy, occupational therapy, cognitive rehabilitation, and structured exercise to improve recovery and independence. In addition, both pharmacologic and non-pharmacologic pain management strategies play an important role in addressing chronic symptoms and supporting rehabilitation efforts. Overall, early recognition of functional deficits and integrated rehabilitation strategies are essential for improving long-term outcomes and quality of life in patients with traumatic brain injury.

## 1. Introduction

Traumatic brain injury (TBI) is defined as a change in brain function and brain pathology due to a force from an external mechanical source, that could have resulted in post-injury loss/alteration of consciousness, post-traumatic amnesia, and potential neurological deficits, without a solid identifiable structural injury or specific signs in neuroimaging [1]. Traumatic brain injury (TBI) is heavily studied due to its effects on public health burden, due to its high incidence rate, wide range of symptoms, and long-term socioeconomic impacts. TBI affects individuals across the lifespan and is a leading cause of death and disability, particularly among children, adolescents, young adults, and older adults [2]. While mild TBI accounts for most cases, mild injuries can result in cognitive decline, emotional and even physical symptoms, as well as the daily lives of patients, employment, and quality of life. More severe effects and higher degree TBIs are more associated with long term effects such as chronic disability, loss of independence, and increased risk of mortality [3].

Classically, TBI injury goes beyond acute effects and can result in a plethora of chronic complications such as post-traumatic headache, neurocognitive impairment, mood and behavioral disorders, sleep disturbances, and epilepsy. These various symptoms contribute to prolonged healthcare utilization, repeated hospitalizations, and long-term reliance on rehabilitation and support services [4]. TBI is also associated with increased risk of neurodegenerative conditions, psychosocial dysfunction, and reduced life expectancy, which in turn can amplify the effects [5].

The most prevalent and disabling long-term consequences for TBIs are functional impairment and chronic pain, and these usually continue well after the immediate recovery period and can debilitate a patien’s lifestyle and decrease quality of life. Functional impairments following TBI are multifactorial and may involve motor, cognitive, sensory, emotional, and behavioral domains, in terms of the initial injury and the long-term consequences [6]. The motor-related deficiencies include generalized weakness, impaired coordination and balance, and gait dysfunction. These motor factors all limit the patients' mobility and limit their daily activity [7]. Cognitive limitations/impairments are also noted, such as limitations in attention, memory, processing speed, and decreased executive function, which can all be impactful in relation to a patien’s ability to work and function in society without being dependent [8]. Even individuals with mild TBI may experience persistent cognitive fatigue and reduced functional efficiency despite minimal findings on neuroimaging.

Pain is one of the most frequently reported chronic symptoms after TBI and represents a major contributor to ongoing disability, where patients frequently complain of post-traumatic headache as the most significant symptom. Post-traumatic headaches often begin as migraine/tension headaches while also exhibiting cervicogenic features. From here, if they persist in a chronic form, they run the risk of creating a compounding effect on the patients alrady existing limitations due to their complexity to treat and how difficult they make participation in other forms of rehabilitation [9]. In addition to headache, individuals with TBI may experience musculoskeletal pain, neuropathic pain, and pain related to spasticity or abnormal muscle tone, particularly in moderate to severe injuries [10]. Due to pain and its multifaceted influence, it can often be exacerbated by compounding factors such as sleep disturbances, mood disorders, and vestibular or cervical dysfunction. This all creates a complex symptom burden that is challenging to treat [11]. The important consensus that can be made is that functional impairment and chronic pain from TBIs are interdependent. Persistent pain plays a large role in limiting engagement in rehabilitation, worsening cognitive performance, and contributing to mood disturbances, while functional limitations and reduced physical activity can amplify pain perception and lead to chronic effects [12]. This interchanging relationship highlights the need for integrated, multidisciplinary approaches that address the issues of impairment and pain simultaneously rather than treating them as individual issues. As a result, long-term outcomes after TBI have not solely been determined by the degree of initial injury, but by how effective the use of coordinated rehabilitation and pain management strategies is/can be through the continuum of care [13].

The issue with initial treatment arises because acute treatment/care prioritizes cervical and is utilized to prevent secondary injury, but this approach causes many individuals to experience persisting functional limitations as well as chronic pain, which drives these patients to long-term disability [14]. This is why the importance of focusing on functional restoration and pain management is essential to improving long-term outcomes after traumatic brain injury (TBI). This is due to these factors playing a defining role in a patien’s independence, participation, and quality of life. Although acute care prioritizes survival and prevention of secondary injury, many individuals experience persistent functional limitations and chronic pain that drive long-term disability and healthcare use [15]. When focusing on functional restoration, the primary targets are to improve mobility, cognition, communication, and executive function that limit return to work, school, and social roles. These outcomes are highly modifiable through timely, task-specific rehabilitation [16]. Pain (post-traumatic headache and musculoskeletal pain) is highly prevalent after TBI and is associated with poorer functional recovery, reduced rehabilitation engagement, and worsened sleep and mood [17]. Due to the correlation of pain and functional impairment, an integrated approach addressing both domains is more beneficial to optimize recovery and achieve more positive outcomes.

The purpose of this literature review is to create a consensus amongst the current evidence on traumatic brain injury (TBI) with a focus on the mechanisms, clinical course, and long-term consequences that influence functional impairment and pain. To dive deeper into this subject, this review will examine the etiology, epidemiology, pathophysiology, disease progression, and associated complications of TBI, while also emphasizing the evidence-based approaches to assessment, rehabilitation, and pain management across the continuum of care. The information will focus on mild, moderate, and severe TBI in adult populations and includes both acute and chronic phases of injury. Due to the studied effects of TBI in terms of long-term recovery, there will be an emphasis on the specific interventions used to target functional restoration and pain control. Emerging and innovative rehabilitation strategies will also be covered, which will ultimately lead to existing gaps in the evidence/knowledge. The goal is to utilize this pertinent information to inform clinical practice and guide future research in TBI management.

## 2. Methods

This narrative literature review synthesizes current evidence on traumatic brain injury (TBI), with particular emphasis on functional impairment, pain, and rehabilitation-based management across the continuum of care. A structured search of PubMed/MEDLINE, Scopus, and Google Scholar was conducted to identify relevant literature. Search terms included combinations of keywords and MeSH terms such as traumatic brain injury, concussion, rehabilitation, functional recovery, cognitive rehabilitation, pain management, post-traumatic headache, epidemiology, and prevention. Reference lists of pertinent review articles and clinical guidelines were also screened to identify additional relevant studies. Articles were included if they were published in English, involved human participants, and addressed one or more of the following domains: epidemiology, pathophysiology, disease trajectory, functional outcomes, pain syndromes, or rehabilitation interventions in TBI. Preference was given to systematic reviews, clinical practice guidelines, randomized controlled trials, and large observational cohort studies to ensure inclusion of high-quality evidence. Studies were excluded if they did not specifically address traumatic brain injury or failed to examine functional or pain-related outcomes. Given the heterogeneity of study designs, populations, and outcome measures across the literature, findings were synthesized qualitatively rather than quantitatively. Results are organized thematically, highlighting areas of consensus, emerging therapeutic strategies, and existing gaps in the evidence base.

## 3. Etiology of Traumatic Brain Injury

**Figure 1:**
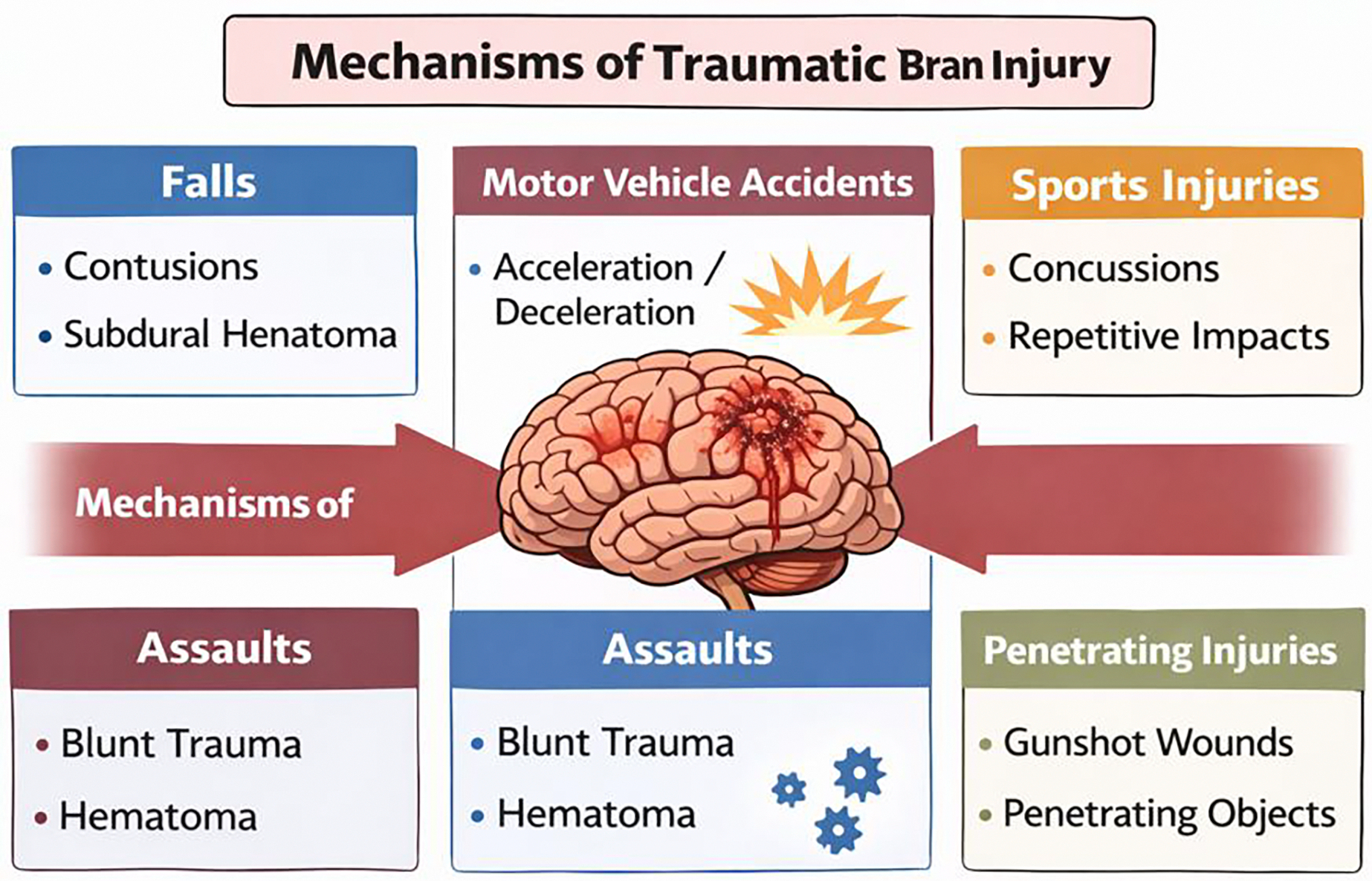
Etiology, Common Sources and Causes of Traumatic Brain Injury

Traumatic brain injury (TBI) is defined as an acquired insult to the brain primarily due to an external mechanical force that may result in temporary/permanent impairment as well as other forms of limitations and pain. The specific mechanism of injury can determine the specific pattern and severity of brain damage, clinical presentation, and recovery trajectory [18]. Concussions are considered mild TBI following a bump, blow, or jolt to the head that causes the brain to move rapidly, disrupting normal function [19]. The leading cause of TBI is blunt head trauma amongst all age groups, which is most caused by falls and motor vehicle accidents (Figure 1). Falls cause injury by creating a rapid deceleration moment on direct impact with a solid surface, which is very likely to cause focal injuries such as cerebral contusions and subdural hematomas [20]. Motor vehicle collisions more commonly generate a rapid acceleration/deceleration moment, which also involves rotational forces that increase risk for diffuse axonal injury, intracranial hemorrhage, and disruption of neural connectivity [21]. Sports/recreational injuries involve either repetitive impact injuries or single impact injuries, both of which have their own rotational components as well. These injuries usually cause concussions and mild TBIs but also result in cumulative injury due to repeated exposure [22]. The rapid change of head velocity may also lead to neuronal dysfunction, metabolic disturbances, and altered neurotransmission as well, even if there is no definitive proof of injury in neuroimaging [23].

Assaults/violence can cause brain injury through blunt force trauma, through physical effects such as punches, kicks, and objects being thrown [24]. Patients who are involved in these types of injuries can face both focal and diffuse damage, and usually also involve facial fractures and intracranial hemorrhages. More severe injuries are caused by repeated assaults/exposure to the mechanism of injury or other high-energy impacts [25]. Blast-related injuries are most seen in intense conflicts and military settings. These injuries are generally more complex due to varying mechanisms and degrees of injury [26]. These are usually caused by overpressure shock waves, massive acceleration/deceleration forces, and traumatic impacts from foreign debris or own bodily displacement [27]. Blast injuries are commonly known to cause a variety of cognitive, emotional, and sensory symptoms due to the associated diffuse brain injury. Penetrating trauma, commonly seen in gunshot wounds and shrapnel injuries, is defined as injuries that penetrate the skull and the dura mater, resulting in injury to the brain tissue inside [28]. Due to the direct injury to neural structures, penetrating brain injury carries a high risk for severe focal neurological deficits, risk for infection and hemorrhage, and poor overall outcomes [29].

In all, reviewing the mechanism of injury is very important to differentiate the varying effects that each mode of injury can have on the brain. Using the mechanism-specific assessments to highlight the concerning effects can aid in providing the appropriate management strategy (Figure 1). Another aspect to consider in TBI is that the pattern of forces that act upon the brain during injury can have different implications for a patien’s recovery and outcome. Linear forces commonly occur when the head is struck or struck by another object, usually resulting from a straight, line acceleration-deceleration-like force [30]. The most common injuries caused by this type of force are focal injuries such as cerebral contusions, skull fractures, and intracranial hemorrhages. The effects of these injuries include neurological deficits and generalized pain in the form of headaches and musculoskeletal pain as well [31]. Rotational forces are the main cause of diffuse axonal injury, which is caused by a shear strain on the brain due to angular acceleration [32]. These types of injuries cause more widespread damage to the brain, such as impaired consciousness, cognitive dysfunction, and prolonged recovery from injury. Even the pain associated with this injury is known to be chronic and due to central nervous system injury, which in turn can be detrimental to sensory processing [33].

Due to different resultant effects, it is important to emphasize the distinction between focal and diffuse injury. When treating focal injuries, they tend to be more predictable because they can be easily visualized on neuroimaging, which can allow for targeted rehabilitation [34]. Diffuse injuries, however, are not always revealed in neuroimaging but are almost always clinically significant, meaning that they can cause a variety of detrimental effects, such as cognitive impairment, behavioral issues, and diffuse pain symptoms [35]. This further explains why it is important to make the distinction between these two forms of injury to create a proper individualized recovery plan to help in mitigating negative chronic effects.

## 4. Epidemiology/Risk Factors/Primary Prevention

### 4.1. Incidence and Prevalence (U.S. and Global)

Traumatic brain injuries (TBI) occur in the United States at an alarming rate, easily exceeding a million cases per year, and globally at tens of millions of cases per year, making it a major public health issue that needs to be addressed on a global scale [36]. Though mild TBI accounts for most of the injuries, the moderate to severe TBI cases are the cases that cause a disproportionate level of mortality, long-term negative chronic effects, and increased healthcare costs. Due to the chronic nature and improved survival rates of these injuries, the global prevalence continues to remain high, affecting the lower/middle-class patients at a higher rate due to varying socioeconomic barriers [37].

### 4.2. Age and Sex Distribution

TBI, like many other prominent forms of injury, displays a bimodal age distribution. This is largely due to the younger populations showing elevated risks due to high involvement in reckless activities such as contact sports, reckless driving, and alcohol related injuries such as falls. In older adults, the elevated risk of TBI is largely due to an increased fall rate and balance issues. Regardless of age grouping, males are typically more likely to experience TBI than females due to the increased exposure to high-risk activities such as dangerous occupations and an increased rate of physical violence [38].

### 4.3. Trends Over Time

TBI incidence has increased over time, mainly due to the improved recognition of injury through imaging and physician training. Even with emergency room visits for TBI’s are increasing, the mortality from these injuries has been consistently decreasing, even in advanced trauma care settings [39]. As infrastructure and car safety have improved, motor vehicle collisions related to TBI are decreasing in incidence, and falls are now the largest contributor, mainly in elderly populations. There are many factors that should be evaluated when referring to TBIs. A prominent factor is alcohol and substance use, which increases the risk for injury due to alcohol's negative effects on a person's senses and increases the risk for injury [40]. Another important factor is environments/occupations, as certain jobs run a higher risk of acquiring head trauma. Finally, social determinants, such as low-income populations, lack of access to healthcare, and unsafe neighborhoods, all play a role in increasing risk for TBI due to a higher predisposition to taking high-risk, unsafe jobs or increased risk for injury from violent altercations [41]. Overall, these factors delineate the multifaceted nature of TBI injury and play an important/overlapping role in prevention and treatment strategies.

When looking for the prevention of TBI, the focus is on reducing the risk of injury, mainly by changing daily behaviors that create a higher risk and altering environmental factors that are within the patien’s control. An example of this would be utilizing a helmet in instances such as riding a motorcycle, scooter, or when playing contact sports, and wearing a seatbelt while driving a car to maximize protection [42]. In elderly groups/young children, it is important to prioritize fall prevention as that is the leading cause of TBI in these populations by providing safety precautions, improving balance and strength through physical therapy/physical activity, and verifying medication side effects. In all, it is important to improve the overall public health awareness of TBI risks, ways to prevent them in public settings, and improve recognition at an earlier timeline. This goes for activities such as contact sports, where it is important promote adequate safety policies to minimize risk for concussion/TBI through repeated exposure to brutal hits to the head, proper protection while on the field, and promoting proper recovery guidelines so that instances of these injuries can decrease [43]. Preventing violence and injuries due to intoxication is through community outreach and intoxication education, which can also greatly decrease the incidence of TBI, as these are very common issues that are being battled in larger cities. When employed together, these different modalities can contribute to decreasing the incidence of TBI across a variety of demographics.

## 5. Pathophysiology of Traumatic Brain Injury

**Figure 2:**
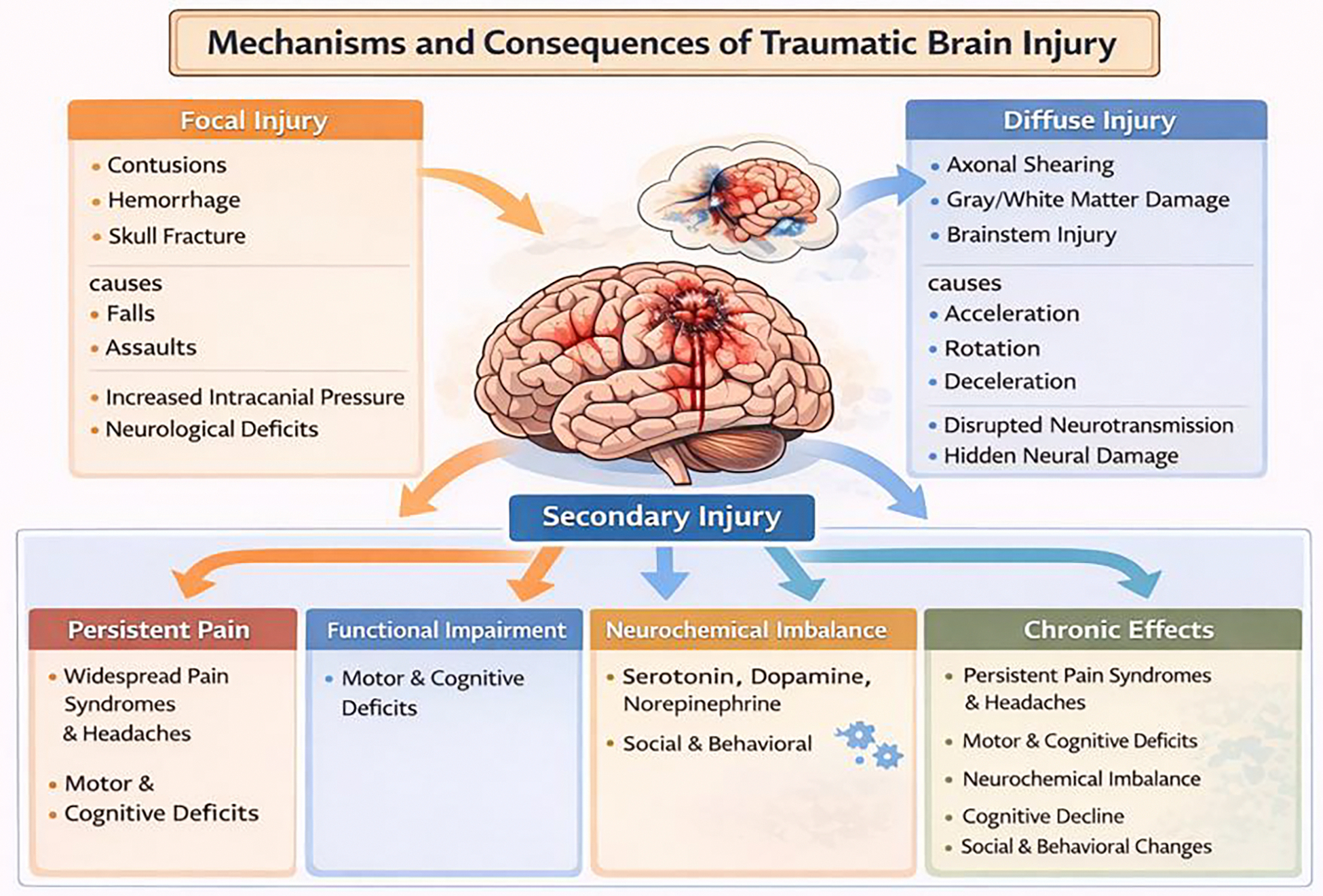
Mechanisms and Consequences of Traumatic Brain Injury

The definition of traumatic brain injury is the initial, irreversible damage to the tissue that is caused by a form of high velocity or repeated impacts, which can be differentiated into focal and diffuse injury types. Ischemia, infarction, and resulting inflammation in the brain due to head injury greatly affect patient outcomes and can be modulated with prompt recognition and treatment [44–47]. Focal injuries result more so from direct contact and the resultant forces produced, which can cause cerebral contusion, hemorrhages, and even fractures to the skull. These symptoms can cause increased intracranial pressure, the creation of masses in the brain, and neurological deficits in certain regions, such as the frontal and temporal lobes (Figure 2). Diffuse injuries, on the other hand, are caused by rotation forces, acceleration, and deceleration forces [48]. These resultant forces cause shearing in the brain in areas of the brain that are commonly affected by car accidents and blast-related injuries, which include the gray/white matter junctions, corpus callosum, and the brainstem. The issue with diffuse injuries, while they disrupt neuronal connections, they do not always show up on neuroimaging, which makes recognizing them difficult [49]. Both focal and diffuse injuries together are characterized by specific recognizable pattern and severity of brain damage, and further study on them can assist with providing proper treatment protocols.

After the original insult, the rise of secondary injuries begins to develop from minutes to days after the initial decision occurs, which contributes to chronic neurological dysfunction. Upon delayed TBI insults, neuroinflammation and excitatory mechanisms are caused by an excess release of excitatory neurotransmitters such as glutamate, which is known to cause depolarization, calcium influx, and further activation of inflammatory pathways through neuronal cells such as astrocytes and microglia [44–47,50]. This delayed effect turns a normally protective inflammatory response into a maladaptive response that causes further degeneration in multiple focal parts of the brain (Figure 2). Another issue is the risk of increased oxidative stress and metabolic crisis caused by deregulation of the mitochondria, which in turn creates an increase in production of antioxidant defense systems, decreased energy production, and disruption of homeostasis [51]. Due to this imbalance, it disrupted neuronal function and continues to lead to the delayed degeneration of certain brain areas. An important deficit to be noted is the increase in intracranial pressure, which creates a disruption of blood flow to across various areas of the brain, which leads to cerebral edema and inhibited autoregulation. Ultimately, due to these effects on decreased cerebral blood flow, ischemia and hypoxia become chronic issues that can be detrimental to the brain tissue. These issues can cause hypotension and hypoxemia, vascular injuries which can lead to further ATP depletion and increased risk for infarction. Though the initial insult can cause extensive damage, secondary/delayed injuries can create even more extensive damage to the brain tissue, leading to chronic issues that are difficult to treat but can be managed with acute management and new/improved neuroprotective strategies [52–55].

The chronic effects of all these cumulative early symptoms result in persistent pain and functional impairment. This is caused by the various pathophysiologic changes in the central nervous system due to the consistent excitatory signaling and increased neuroinflammation. These all play a part in increasing the pain signaling pathways, which cause the chronic pain. This can result in widespread pain syndromes, headaches, and even neuropathic pain [56]. Many patients experiencing these chronic issues report pain levels at debilitating levels, resulting in functional impairment. When looking at the social impacts of TBI, it is important to discuss the altered neuronal connections caused by the dysfunction that impairs communication between the cortical and subcortical networks, which in turn affects motor control, sensory processing, and cognition. Diffuse injury can cause further damage, leading to deficits in mobility, coordination, and even executive function, which compound a patien’s functional pain [57]. Imbalances of neurotransmitters are also critical in the analysis of both neurochemical and autonomic dysregulation. These neurotransmitters play crucial roles in pain modulation, mood, sleep, and motivation, which are imperative in recovery and continuation in progressive rehabilitation. Without correction of these imbalances, it can lead to abnormal nervous system responses such as headaches, fatigue, and stress reactivity [58]. When put together, the mechanisms of this pathophysiology explain why TBI results in chronic pain and a plethora of limitations that can potentially be treated with targeted rehabilitation.

## 6. Disease progression/Disease trajectory

Following the progression is an important study due to TBI showing overlapping clinical symptoms throughout the different stages of this process. Through the different phases, you will see the various neurological deficits, altered need for rehabilitation, and further complications that patients tend to experience. Starting with the acute phase (hours-days), shows initial altered consciousness, depending on whether it is focal/diffuse neurological deficits, and early complications that have been stated above, such as increased intracranial pressure, cerebral edema, and seizures [59]. The earlier these symptoms are treated, the less likely they are to progress to secondary injury. Now onto the subacute phase (days-weeks), this is where symptoms such as amnesia and decreased awareness become prominent, which also lead to behavioral and cognitive impairment. This tends to be the ideal time for large-scale rehabilitation to prevent chronic issues [60]. Finally, the post-acute stage (weeks to months) is where patients will show the most impairment and will be experiencing the chronic symptoms such as reduced functionality and chronic pain, such as headaches, fatigue, and sleep disturbances. Now, depending on the severity of the injury, this stage is where the patients will also demonstrate the most functional recovery if they consistently attend and participate in intense rehabilitation. Now, if rehabilitation regimens are not followed, patients fall into the chronic section (months-years) where patients deal with long-term disability that tends to affect all aspects of their life [61]. Recovery at this period of the injury is mainly just preserving function that is left due to the chronic symptoms, causing cognitive dysfunction and neuropsychiatric issues as well.

Determining the trajectory of the disease is difficult as it is progressive and interrelated across a plethora of different domains, such as motor, cognitive, and even emotional. Commonly, motor deficits and cognitive limitations are the first symptoms seen and the ones that cause the patient the most issues. These limitations include disturbances in gait, balance, and mobility for movement, and limitations in attention, memory, and problem-solving for cognitive issues. Behavioral and emotional concerns also arise due to limitations and degeneration of certain neuronal pathways, causing symptoms such as depression, anxiety, and decreased social functioning [62]. Now, when these injuries become chronic, pain syndromes have a serious effect on a patien’s life. They create sleep disturbances, mood changes, and, when put together with all the other symptoms, creates difficult in daily activities and decrease a patien’s quality of life.

## 7. Associated Conditions/Complications

TBI can also cause a plethora of associated conditions that can stem from the injury course and can significantly affect recovery and treatment outcomes. Many of these symptoms have been discussed above such as post-traumatic headaches, sleep disorders, and mood/substance use disorders, but there are other ones to discuss, such as post-traumatic epilepsy, which mainly occurs with moderate to severe TBIs but can carry long-term risk for future injury and impairment of cognition [63]. Another is neuroendocrine disorders, such as pituitary hormone dysfunction, which can also lead to a lot of the associated symptoms that these patients face, such as decreased energy, hypogonadism, and impaired cognition. In addition, in severe cases, dysphagia and nutritional deficits can occur due to cranial nerve damage and reflex inhibition, which causes an increased risk of aspiration and malnutrition. Ultimately, the most drastic symptoms are complex TBIs, causing early morbidity. The best way to treat these is early diagnosis, continuous monitoring, and actively attending rehabilitation sessions with active participation to improve independence and quality of life [64].

## 8. Injury Assessment

To accurately assess traumatic brain injury, there are multiple different tools that can be used to determine prognostics, management, and potential rehabilitation plans. To rate the injury severity, physicians tend to use the Glasgow Coma Scale, which allows a TBI to be rated as a mild, moderate, or severe level injury [65]. Neuroimaging, on the other hand, can be used to locate hemorrhages, contusions, and fractures that occurred due to the initial injury [66]. More importantly, to assess amnesia, cognition, and physical function, physicians use different scales such as the Rancho Los Amigos levels of cognitive functioning, disability rating scale, and the functional independence measure. It is imperative that a proper assessment is made because it will likely determine the course of the patien’s rehabilitation and that their various symptoms are properly addressed [67]. This would all result in a holistic understanding of what the patien’s symptoms are and how they can be reduced in severity, increase functional capacity, and prevent further harm from coming to the patient.

## 9. Treatment and Rehabilitation Management

Treatment for TBI varies largely based on the degree of injury. There is no effective therapeutic strategy targeting neuronal injury and neuroinflammation in TBI to improve neurological deficits and cognitive impairment. The current paradigm in TBI management and treatment hinges on the protection of the neural elements from secondary injury. However, most of the clinical trials of TBI pharmaceutical agents for the diagnosis and/or treatment have been unsuccessful. Nonetheless, several novel treatment strategies are currently under investigation. These include innovative neurostimulation treatments, including non-invasively with electromagnetic field stimulation that modulate inflammatory response in neuronal and microglial cells and phytochemicals [68–77]. Recent pre-clinical and clinical studies have shown significant improvement in the healing of brain tissue with functional recovery following traumatic brain injury [53–55].

**Figure 3:**
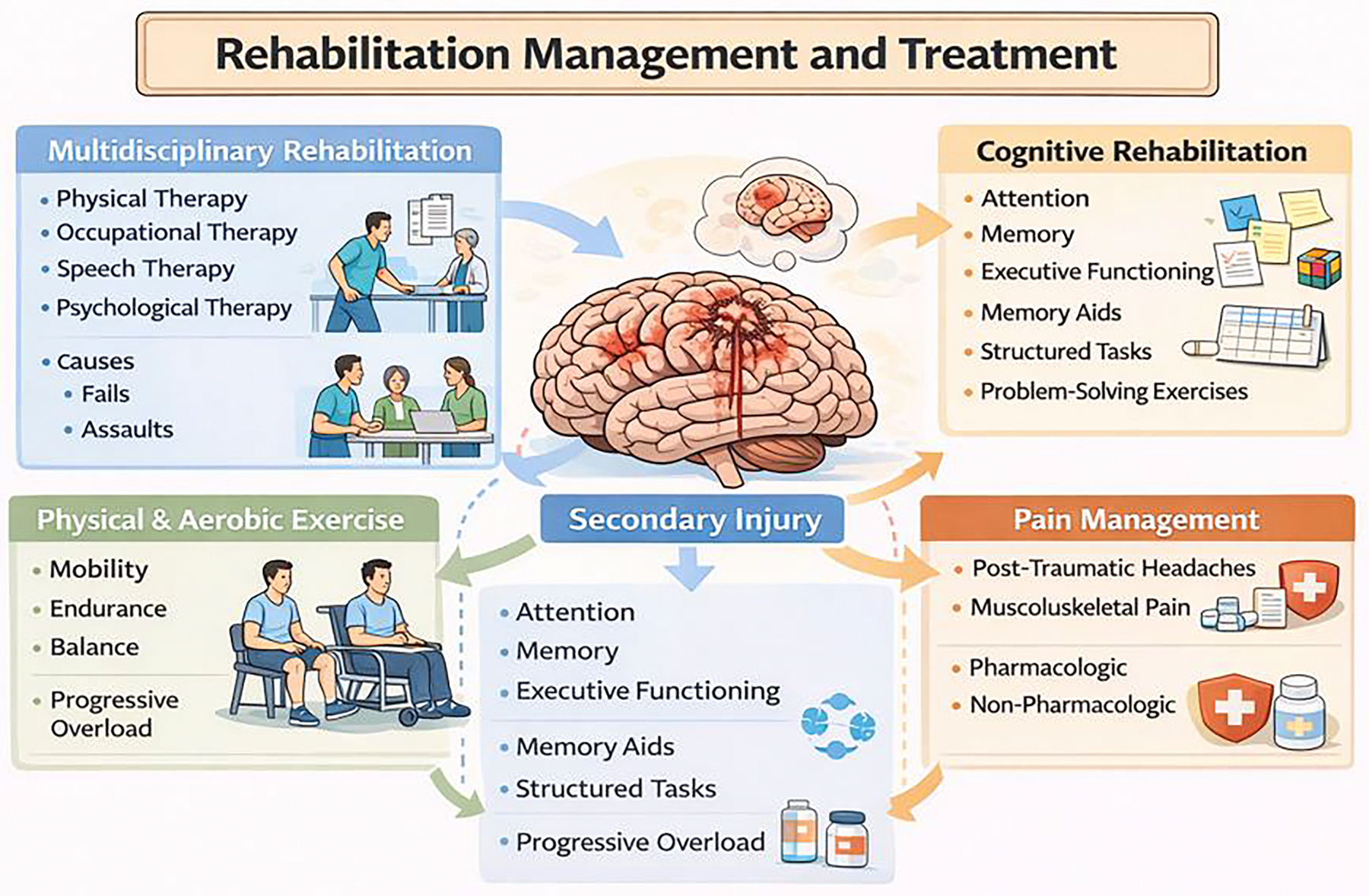
Rehabilitation Management and Treatment of Traumatic Brain Injury

Targeted rehabilitation is considered the most effective treatment through individualized approach that targets specific issues that patients are facing. Multidisciplinary rehabilitation is known as the coordinated care of various modalities such as physical therapy, occupational therapy, speech therapy, and consistent psychological evaluations [78]. Each one of these therapies targets a specific impairment and works to get the patient back to functional capabilities. Patient tends to focus on mobility and balance, occupational therapy works to improve daily lifestyle activities, speech therapy is utilized to regain any linguistic capabilities that were lost, and continuous psychological evaluation is conducted to make sure the patient is not declining mentally [79]. This form of treatment is only beneficial when patients keep up rehabilitation intensity and continuously attend sessions to improve neuroplasticity and to undergo maximum recovery. Pressure injuries could also be prevented in patients with impaired mobility [80]. Next would be to address any cognitive losses with cognitive rehabilitation, which mainly targets attention, memory, and executive functioning. These can be targeted mainly through a strategic approach that includes memory aids, performing tasks in specific orders, and performing problem-solving exercises [81]. The main goal for this therapy is to improve a patients real world cognition and allow them to get back into daily life without too much limitation (Figure 3).

Another form of rehabilitation is physical and aerobic exercise intervention that can be used to improve mobility, endurance, and balance. These interventions are usually led by physical therapists to include progressive overload so that the patient continues to improve over time, improve gait, balance, and aerobic exercise [82]. Improvement in these factors has been shown to improve post-concussive symptoms, improve cognition, and improve the patien’s physical capabilities. One of the important aspects that is beneficial for patients is pain management. The most prominent symptoms that are treated are post traumatic headaches and musculoskeletal pain. Primarily, the initial treatment is pharmacological approaches, such as analgesics, non-pharmacological interventions, such as further therapy, osteopathic approaches, and mindfulness-based techniques that can also help improve a patien’s outcome [83].

When treating severe TBI, it is very important to apply early innervations which could include pharmacologic management of cognitive disorders, which can be used in combination with both acute and subacute rehabilitation strategies. This coordination of care is beneficial to promote functional recovery, prevent further secondary complications, and even support a patien’s reintegration into society, whether it be school, work, or just daily life [84]. In conclusion, rehabilitation and additional treatment strategies really emphasize a holistic approach to TBIs and provide patient-centered methods to approach the various physical, cognitive, and pain-related symptoms that patients experience (Figure 3).

## 10. Future Directions

Currently, there are a plethora of new modalities that can be used to treat traumatic brain injuries and enhance recovery through technology-driven approaches that can be individualized to a patien’s needs. A new treatment that is being preferred for patients is Noninvasive brain stimulation, which mainly aims to modulate cortical excitement and even improve motor control and cognition [85]. In addition, new research states that using new technology, such as virtual reality and robot-assisted rehabilitation, provides patients with immersive and interactive environments that support their specific issues, improve functional capabilities, and offer real-time feedback [86]. To prevent a lack of participation in rehabilitation, tele-rehabilitation and remote monitoring are becoming more popular methods to allow therapy in an at-home setting through wearable devices and digital markers that can be used to track movements to optimize a patien’s treatment and allow for easy access as well [87]. All these different models use precision rehabilitation-related models that integrate various information, such as clinical information, neuroimaging, and behavioral data, to optimally track progress and utilize this information to provide data-driven, individualized TBI care.

## 11. Gaps in Knowledge

Even with all the technological advancements and a bright future in TBI treatment, there are still plenty of gaps in knowledge that remain in the evidence base that still limit the treatment strategies for patients. One major issue is the vast range of injuries in TBI populations. The necessity for individualized patient care is a way to bridge this gap, but there are vast variations in relation to a patien’s injury severity, mechanism of injury, age, and different comorbidities that could be present based on an individual patien’s lifestyle habits. These various factors tend to complicate patient comparison to others with similar injuries and drastically reduce the generalizability of both treatment and future research on this subject. In relation to this, the variability of these types of injuries also increased the variability in measuring outcome and interventional dosing, further propagating the use of individualized models rather than generalized treatments [88]. This makes it more difficult to determine optimal treatment parameters in patients due to differences in functional capabilities, cognitive abilities, and intensity of rehabilitation.

When it comes to studying the effects of TBI and its rehabilitation course, there are a limited number of long-term randomized clinical trials, which makes it difficult to form a proper understanding of the functional recovery and chronic pain treatment trajectory. The current issues that a plethora of studies face are that they mainly tend to focus on discrete cognitive, motor, and behavioral factors individually, which tends to lead to an insufficient integration of pain management and other functional outcomes, which propagates an poor overall recovery and a decreased quality of life [89]. Finally, the limitations that are most commonly faces is the barrier to implementation, which occurs due to limited access to specialized rehabilitation services, resources constraint, and a variability of clinician training, which all play strong factors in hindering translation of evidence into routine practice [90]. Addressing these limitations will require standardized outcome measures, robust trial designs, and implementation of new and upcoming research to improve rehabilitation and create better outcomes.

## Conclusion

Indeed, traumatic brain injury is a complex, multisystem injury that creates multiple issues in patients, such as functional, cognitive, and pain-related components. In terms of treatment, it is beneficial to provide early, individualized rehabilitation and a personal pain management regimen to create an optimal plan for patients to optimize recovery, enhance independence, and improve overall quality of life. With new technologies arising, it is becoming increasingly possible to enhance patient outcomes, but gaps in our knowledge remain. Addressing these gaps through new treatments and research methods, such as the use of patient-centered research and new implementation strategies, will work to advance evidence-based care and to improve long term outcomes for patients living with TBI.

## Key Points

- Traumatic brain injury (TBI) is a major public health issue and a leading cause of death and long-term disability worldwide.
- Common causes of TBI include falls, motor vehicle accidents, sports injuries, assaults, and penetrating trauma.
- TBI can produce focal injuries (contusions, hemorrhage) or diffuse injuries (diffuse axonal injury) that disrupt neural connectivity.
- Secondary injury processes such as neuroinflammation, excitotoxicity, and oxidative stress contribute to long-term damage.
- Functional impairment after TBI often includes motor deficits, cognitive dysfunction, and impaired balance or coordination.
- Chronic pain is common after TBI, particularly post-traumatic headaches and musculoskeletal pain.
- Pain and functional impairment are closely linked, often worsening rehabilitation outcomes and quality of life.
- Multidisciplinary rehabilitation is essential, incorporating physical, occupational, speech, and psychological therapies.
- Cognitive rehabilitation and aerobic exercise can improve cognitive function and physical recovery.
- Emerging technologies such as virtual reality and tele-rehabilitation may enhance recovery, though further research is needed.
